# Early detection of COVID-19 cholangiopathy using cholangioscopy—a case report of two critically ill patients

**DOI:** 10.1007/s10353-022-00776-6

**Published:** 2022-09-23

**Authors:** Veronika Kroepfl, Benedikt Treml, Martin Cornelius Freund, Christoph Profanter

**Affiliations:** 1 Department of Visceral, Transplant and Thoracic Surgery, Center of Operative Medicine, Medical University of Innsbruck, Anichstr. 35, 6020 Innsbruck, Austria; 2 General and Surgical Intensive Care Unit, Department of Anesthesiology and Critical Care Medicine, Medical University of Innsbruck, Anichstr. 35, 6020 Innsbruck, Austria; 3 Department of Radiology, Medical University of Innsbruck, Anichstr. 35, 6020 Innsbruck, Austria

**Keywords:** COVID-19 associated cholangiopathy, Hepathopathy, Secondary sclerosing cholangitis, COVID-19 gastrointestinal manifestations, Intensive-care related complications

## Abstract

**Background:**

The coronavirus disease 2019 (COVID-19) crisis caused by the severe respiratory distress syndrome coronavirus 2 (SARS-CoV-2) rapidly led to a pandemic. While the majority of SARS-CoV-2-infected patients present with fever and respiratory symptoms, gastrointestinal symptoms may also occur. In addition, serious hepatic manifestations like cholangiopathy and liver failure have been described.

**Patients and methods:**

We identified two critically ill patients suffering from SARS-CoV‑2 infection in our intensive care unit (ICU). In both patients, laboratory testing revealed elevated liver chemistries weeks after initial diagnosis with COVID-19.

**Results:**

During repeated endoscopic retrograde cholangiopancreatography (ERCP) with cholangioscopy, a severely destructed biliary mucosa with ischemia and epithelial roughness was seen in both patients. Due to the prolonged course of COVID-19 and chronic liver damage with ongoing sepsis, both patients succumbed to the disease.

**Conclusion:**

In our opinion, a COVID-19 infection can lead to development of cholangiopathy in critically ill patients. Cholangioscopy performed early can confirm the diagnosis of COVID-19-associated cholangioscopy.

## Introduction

Coronavirus disease 2019 (COVID-19) emerged for the first time in December 2019 and rapidly spread all over the world, necessitating its declaration as a pandemic by the World Health Organization [1]. As pneumonia is the main feature of this disease, other organ systems like the digestive system may be affected as well [2].

Impaired liver function tests and liver injury are described, ranging from mild to severe [3]. More serious hepatic affections with cholangiopathy, liver failure, and development into secondary biliary cirrhosis have been described recently as a complication of severe COVID-19 infection occurring several months after initial diagnosis [4, 5]. Cholangiocytes express angiotensin-converting enzyme 2 (ACE) receptor, which may be seen as the hepatic entry point of the virus [6]. This may cause direct cytopathic damage of the cholangiocytes, resulting in chronic liver damage and, finally, in secondary biliary cirrhosis [7].

Here we describe our cholangioscopic findings and the clinical course of two critically ill patients suffering from severe COVID-19-associated cholangiopathy.

## Patients and methods

Endoscopic procedures—endoscopic retrograde cholangiopancreatography (ERCP) and cholangioscopy—were performed under general anesthesia using the following equipment:

- Duodenoscope TJF‑Q 180V, TJF‑Q 190V, and processor EVIS EXERA III CV-190 (Olympus K. K., Shinjuku, Tokyo, Japan)
- Sphincterotome Clever Cut 3V (Olympus)
- Balloon catheter Multi-3 V Plus (Olympus)
- Spy Glass DS II (Boston Scientific, Marlborough, MA, USA)
- Guidewire 0.035 inch × 450 cm (Boston Scientific)
- CO_2_ insufflator from Olympus (Olympus)
- Digital fluoroscopy systems: AXIOM Luminos dRF (Siemens, Munich, Germany) and Clarity Monoplan (Philips Medical Systems, Hamburg, Germany)

We identified two critically ill male adults suffering from an acute respiratory distress syndrome (ARDS) as part of a COVID-19 infection. The past medical history included bronchial asthma, hepatic steatosis, and monoclonal gammopathy of undetermined significance (MGUS), to mention the most important.

## Results

**Fig. 1:**
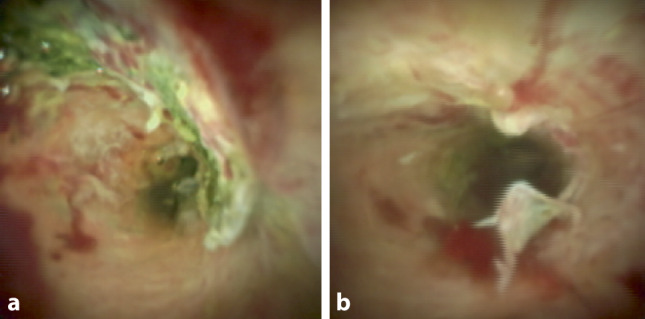
Images **a** and **b** show cholangioscopic views after sludge and cast removal in patient 1: the biliary mucosa appeared severely damaged with small hematomas, roughness, and hyperemia

Due to the severe ARDS and superinfection with *Klebsiella pneumonia* and *Aspergillus fumigatus*, patient 1 required venovenous extracorporeal membrane oxygenation (vvECMO). Moreover, COVID-19-associated lung failure necessitated bilateral lung transplantation 85 days after COVID-19 infection. During the procedure, iatrogenic perforation of a right-sided esophageal diverticulum occurred. The lesion was surgically secured. Postoperatively on day 17 after transplantation, due to distinctly increased cholestasis markers, ERCP was performed. Beside removal of multiple biliary stones and sludge using a balloon catheter, the mucosa of the biliary system appeared severely destructed with isolated small hematomas, epithelial roughness, and hyperemia (Fig. 1).

Septic shock due to esophageal perforation after endoscopy occurred, requiring several surgical procedures due to a thoracic infection.

**Fig. 2:**
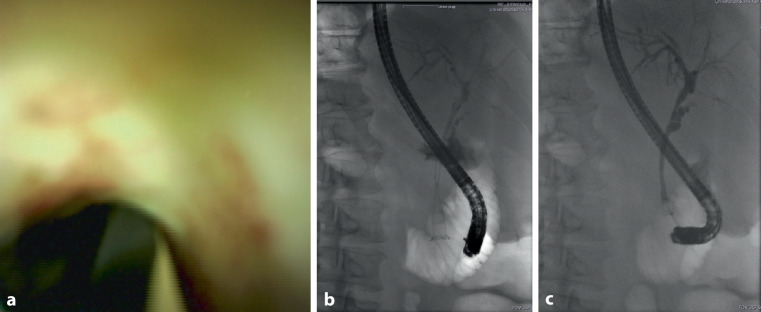
Similar to patient 1, cholangioscopy in patient 2 showed an irregular and ischemic pattern of the biliary mucosa (**a**); after stent-implantation, the common hepatic duct did not expand more than 4 mm (**b**, **c**)

Patient 2 was admitted from a primary care hospital due to lung failure and for evaluation of ECMO therapy. The latter was refrained from as a consequence of a prolonged course of COVID-19 and a limited prognosis regarding rehabilitation. At the time of admission, liver chemistries were already elevated. Imaging and ERCP with cholangioscopy displayed ischemic cholangiopathy similar to patient 1. A fully covered self-expanding Wallstent (Wallflex, Boston Scientific, diameter 10 mm, length 80 mm) had to be inserted to stop diffuse bleeding of the major duodenal papilla 3 weeks after initial ERCP. The stent did not expand to more than 4 mm (Fig. 2).

**Fig. 3:**
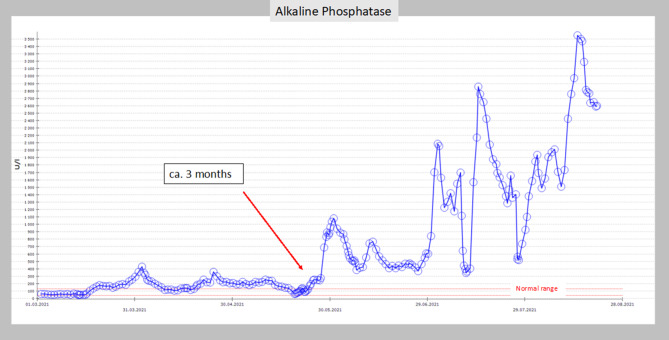
Timeline and course of liver parameters (alkaline phosphatase) as seen in patient 1

**Fig. 4:**
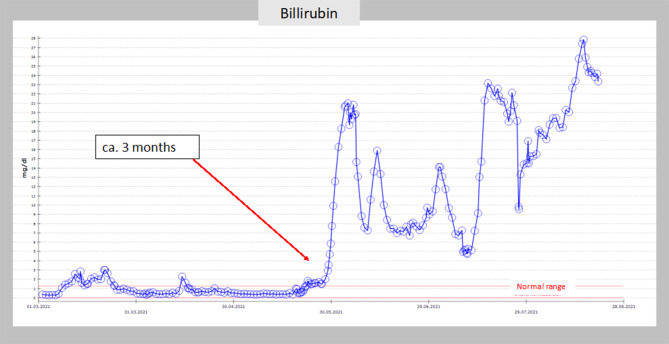
Timeline and course of liver parameters (bilirubin) as seen in patient 1

In both patients, we found the same pattern of changes in laboratory testing of the liver chemistries: 2.5 months after initial diagnosis of COVID-19, aspartate aminotranferase (AST) and alanine aminotransferase (ALT) first increased up to 1000–1200 mg/dl; after this peak, 2 weeks later, the cholestatic parameters increased, with a bilirubin level up to 20 mg/dl and alkaline phosphatase (ALP) up to 1200 mg/dl in patient 1 and patient 2 (Figs. 3 and 4).

**Fig. 5:**
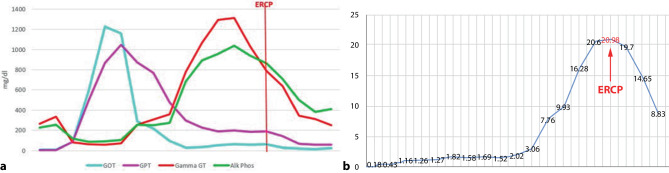
Course of liver parameters in patient 1: first, we observed an increase of transaminases, followed by an increase of ALP/gamma glutamyltransferase (gamma GT) (**a**). ERCP was performed at time of bilirubin elevation (**b**). *GOT* AST, *GPT* ALT, *Alk Phos* ALP

During the ICU stay, cholestatic parameters increased further and repeated ERCP with cholangioscopy revealed an irregular biliary mucosa as a sign of ischemic cholangiopathy. This pattern resembles the cholangioscopic findings in patients with ischemic damage of arterial perfusion after liver transplantation.

Given the compulsory respiratory support, chronic liver damage, and ongoing sepsis, the prognosis was expected to be unfavorable. Thus, further treatment was withdrawn after reaching consensus with next of kin. Both patients succumbed to their disease.

## Discussion

Recently, cholangiopathy in patients with severe COVID-19 infection has been described as a new entity [4, 5, 7]. Providing unique histological features, so-called COVID-associated cholangiopathy or post-COVID cholangiopathy has to be differentiated from secondary sclerosing cholangitis in critically ill patients (SSC-CIP) [7, 8]. We performed ERCP and cholangioscopy at the time of bilirubin elevation.

We found notable roughness of the biliary epithelium, signs of inflammation (hyperemia), and lesions like small intramural hematomas in the extrahepatic bile ducts. Radiologic ERCP findings showed no strictures and no bile duct rarefication in either patient, whereas other features were different: in patient 1, the common hepatic duct (CHD) was only slightly dilated (about 13 mm) with normal intrahepatic bile ducts. The biliary system was cleared from blood and cast. Patient 2 had no blood or cast, but the bile ducts appeared uncommonly narrow with a CHD diameter of only 4 mm. We think this condition was due to severe inflammation.

Our findings, with changes of the laboratory pattern and the timeline, correspond exactly to the findings in the literature. ALP and bilirubin were the most striking features of the liver parameters in the COVID-19 patients [9]. Thus, we believe that cholangioscopic findings in our two patients are due to COVID-19 associated cholangiopathy.

Given the scarcity of literature, we want to add some considerations regarding the significance of cholangioscopy in patients with COVID-19 cholangiopathy. The question remains of whether pathological changes of the biliary epithelium itself occur several weeks before cholangiopathy becomes visible in radiological imaging like MRCP or fluoroscopy. Regarding the similarity of cholangioscopic findings in transplant patients and COVID-19 patients, it is our opinion that cholangioscopy could lead to early detection of COVID-19-associated cholangiopathy after clinical suspicion.

The underlying cause of COVID-19-associated cholangiopathy is still unknown. Moreover, as we cannot rule out a contributing effect of ischemic hepatopathy, a multifactorial cause seems at least possible. Clearly, further studies will have to shed light on such association. Even if a recently discussed direct cytopathic effect proves to be correct, a medical treatment to stop or slow the progression of COVID-19-induced cholangiopathy does not currently exist. Moreover, these patients are likely candidates for liver transplantation in the future. It is still unknown how many patients after severe COVID-19 infection will develop such a cholangiopathy. Given the incidence of COVID-19, even a small percentage could mean a great many. Therefore, early diagnosis could help to identify patients developing secondary biliary cirrhosis after COVID-19 infection. This may tailor further medical treatment, at least for preventing futile therapy.

## Conclusion

In summary, we recommend ERCP with cholangioscopy in case of clinical signs of cholestasis at an early stage in patients with a severe course of COVID-19. In our opinion, the sooner this procedure is performed, the sooner cholangioscopy may detect COVID-19-associated cholangiopathy. However, without treatment for these patients at hand, the focus of ERCP with cholangioscopy will rather be prediction of outcome. Clearly, further investigations are warranted.
